# Editorial

**Published:** 1866-07

**Authors:** 


MORTALITY FOR THE MONTH OF APRIL.—The mortality report for the month of April, as compiled by the health-officer, is given below. The report shows an increase of 28 deaths over the mortality of the same month in 1865, and an increase of 24 over the number for March. It would be difficult for the disciple of Hippocrates, however, metaphysically inclined, to deduce from the following careful figures any indications of a visitation of the cholera. The number as usual will increase until September, consequent on the usual summer causes:—

CAUSES OF DEATH.

| | | | |
|---|---|---|---|
| Accidents, | 7 | Fever, Scarlet, | 6 |
| Bright's Disease, | 1 | Fever, Typhoid, | 9 |
| Bronchitis, | 1 | Fever, Typhus, | 2 |
| Burned, | 2 | Hydrocephalus, | 6 |
| Cancer, | 2 | Inanition | 1 |
| Childbed, | 8 | Inflammation of Brain, | 11 |
| Chlorosis, | 1 | Inflammation of Bowels, | 8 |
| Colic, | 1 | Inflammation of Lungs, | 24 |
| Congestion of Brain, | 4 | Inflammation of Throat, | 1 |
| Congestion of Lungs, | 4 | Intemperance, | 1 |
| Consumption, | 37 | Measles, | 4 |
| Convulsions, | 23 | Neuralgia, | 1 |
| Croup, | 16 | Old Age, | 9 |
| Debility, | 1 | Poisoning, | 1 |
| Delirium Tremens, | 1 | Rheumatism, | 2 |
| Diarrhœa, | 2 | Scrofula, | 1 |
| Diarrhœa, Chronic, | 1 | Stillborn, | 9 |
| Diphtheria, | 5 | Stone in Bladder, | 1 |
| Disease of Bowels, | 2 | Suffocation, | 2 |
| Disease of Heart, | 1 | Teething, | 5 |
| Disease of Liver, | 3 | Tuberculosis, | 1 |
| Dropsy, | 9 | Typhoid Pneumonia, | 1 |
| Drowned, | 6 | Unknown, | 23 |
| Dysentery, | 3 | Whooping-Cough, | 1 |
| Erysipelas, | 1 | Wound, | 2 |
| Fever, Childbed, | 2 | | |
| Fever, Congestive, | 1 | Total, | 278 |
| Fever, Remittent, | 1 | | |

AGES OF THE DECEASED.—Under 5 years, 129; over 5 and under 10 years, 16; over 10 and under 20, 17; over 20 and under 30, 36; over 30 and under 40, 23; over 40 and under 50, 24; over 50 and under 60, 11; over 60 and under 70, 8; over 70 and under 80, 6; over 80 and under 90, 1; unknown, 7. Total, 278.

NATIVITIES.

| | | | | | |
|---|---|---|---|---|---|
| Chicago, | 128 | France, | 1 | Sweden, | 2 |
| Other States, | 56 | Holland, | 1 | Unknown, | 6 |
| Canada, | 2 | Ireland, | 24 | | |
| Denmark, | 1 | Norway, | 4 | Total, | 278 |
| England, | 3 | Nova Scotia | 1 | | |
| Germany, | 45 | Scotland, | 4 | | |

| | | | | | | | |
|---|---|---|---|---|---|---|---|
| North, | 65 | South, | 99 | West, | 114 | Total, | 278 |

EXPLANATION.—Several editorial articles and book notices are crowded out of this number by the Proceedings of the Illinois State Medical Society.

PERSONAL.—We learn with pleasure that our esteemed friend Dr. LOUIS BAUER, of Brooklyn, N.Y., has returned from his recent visit to Europe, as vigorous in health and professional enthusiasm as ever.

